# An illustration of and programs estimating attributable fractions in large scale surveys considering multiple risk factors

**DOI:** 10.1186/1471-2288-9-7

**Published:** 2009-01-23

**Authors:** Simon Rückinger, Rüdiger von Kries, André Michael Toschke

**Affiliations:** 1 Ludwig-Maximilians-University of Munich, Institute of Social Pediatrics and Adolescent Medicine, Division of Epidemiology, Munich, Germany; 2 King's College London, Division of Health and Social Care Research, Department of Public Health Sciences, London, UK

## Abstract

**Background:**

Attributable fractions (AF) assess the proportion of cases in a population attributable to certain risk factors but are infrequently reported and mostly calculated without considering potential confounders. While logistic regression for adjusted individual estimates of odds ratios (OR) is widely used, similar approaches for AFs are rarely applied.

**Methods:**

Different methods for calculating adjusted AFs to risk factors of cardiovascular disease (CVD) were applied using data from the National Health and Nutrition Examination Survey (NHANES). We compared AFs from the unadjusted approach using Levin's formula, from Levin's formula using adjusted OR estimates, from logistic regression according to Bruzzi's approach, from logistic regression with sequential removal of risk factors ('sequential AF') and from logistic regression with all possible removal sequences and subsequent averaging ('average AF').

**Results:**

AFs following the unadjusted and adjusted (using adjusted ORs) Levin's approach yielded clearly higher estimates with a total sum of more than 100% compared to adjusted approaches with sums < 100%. Since AFs from logistic regression were related to the removal sequence of risk factors, all possible sequences were considered and estimates were averaged. These average AFs yielded plausible estimates of the population impact of considered risk factors on CVD with a total sum of 90%. The average AFs for total and HDL cholesterol levels were 17%, for hypertension 16%, for smoking 11%, and for diabetes 5%.

**Conclusion:**

Average AFs provide plausible estimates of population attributable risks and should therefore be reported at least to supplement unadjusted estimates. We provide functions/macros for commonly used statistical programs to encourage other researchers to calculate and report average AFs.

## Background

The major burden of disease has shifted from communicable to non-communicable diseases in high-income countries during the past century [1,2]. Populations are aging in most high income countries, resulting in a further increase of non-communicable diseases [3]. This accumulation of prevalent non-communicable diseases and their sequelae represent a major challenge for health service capacities and financial resources. Policy makers need evidence based advice for decisions on potential interventions and population based prevention strategies.

While publications often report estimates of individual associations such as relative risks or odds ratios, attributable fractions (AFs) are infrequently reported. The AF quantifies the proportion of cases that can be attributed to a certain risk factor for a specific disease, for example, the proportion of lung cancer cases attributable to smoking. Smokers have a highly increased risk of lung cancer. However, this individual risk does not give any information on the relevance of smoking for lung cancer in a population also containing nonsmokers. AFs help assessing a potential impact of preventive interventions on population health.

A number of risk factors for non-communicable diseases have been established such as hypertension for cardiovascular disease [2]. Multivariable logistic regression has become a standard procedure to provide valid estimates of individual risk studies. Similar methods, however, have rarely been applied for AFs, although corresponding approaches have been described before [4-6]. Their infrequent application in public health research might particularly be due to lacking inclusion of their estimates in statistical software packages.

The main aim of this paper is to illustrate the use of AFs by comparing different approaches estimating AFs. Therefore we used established risk factors of cardiovascular disease in the 2005–2006 National Health and Nutrition Examination Survey (NHANES) for illustration purposes [7]. Additionally, we provide functions/macros for the frequently used statistical software packages R [8], SAS [9], and STATA [10], allowing readers to recalculate the results shown and moreover, to encourage readers to calculate and report adjusted AFs of their own research observations.

## Methods

### Definition of the attributable risk

Throughout this paper, we refer to the attributable fraction (AF). A risk factor strongly associated with the disease, but infrequently prevalent in the population, is less relevant compared with a risk factor of similar effect magnitude affecting a larger proportion of the population. The AF considers both, the individual association and the exposure frequency and thus, allows to estimate the relevance of a risk factor for a disease in a population. The definition of AFs used in this paper reflects the proportion of cases that can be attributed to a certain risk factor in a population.

### Levin's formula

One of the most frequently applied approaches calculating the AF is the Levin formula. It is named after its first describer who introduced the concept of calculating attributable risks in 1953 [11]. The idea is to separate the number of cases into expected and excess cases. The expected cases are calculated under the assumption that the proportion of cases should be equal among the exposed and unexposed. The cases among the exposed exceeding the expected number of cases based on the estimate derived from the prevalence of the disease among the unexposed are supposed to be cases attributable to the risk factor. Based on this assumption Levin described a formula that requires only the relative risk estimate (*RR*) and the prevalence of the risk factor (*p*):

$$AF_{Levin}=\frac{p\cdot (RR-1)}{1+p\cdot (RR-1)}$$

The relative risk is often approximated by the odds ratio e.g. in cross-sectional studies.

### Plug-in and Bruzzi's method

One approach sometimes used to adjust AFs for other known risk factors considers adjusted odds ratio estimates from multivariable logistic regression analysis in Levin's formula [12,13]. This approach has a couple of disadvantages as we outline in the discussion section. Another approach using logistic regression estimates was suggested by Bruzzi et al [14]. This method provides adjusted AFs and was originally presented for case-control data but can also be applied in cross-sectional studies.

### Sequential and average AF

The concept of obtaining AFs directly from logistic regression was introduced by Greenland and Drescher [15]. The basic idea behind this approach is to estimate a logistic regression model with all known/available risk factors. The AF of the risk factor of interest is then calculated as follows:

1. The risk factor has to be coded dichotomously. It is 'removed' from the population by classifying all individuals as unexposed, irrespective of their real status.

2. A logistic model using this modified dataset is used to estimate predicted probabilities for each individual:

$$pp_{i}=\frac{1}{1+\exp(-(\alpha +\beta 'x_{i}))}$$

where α represents the estimate for the intercept of the logistic regression model, β denotes the parameter vector for the covariates included in the model, and *x*_*i *_denoting the observations of the covariates for each individual, however, with the 'removed' covariate set to zero for all individuals.

3. The sum of all predicted probabilities is the adjusted number of cases of the disease that would be expected if the risk factor was absent in the population.

4. The AF is then calculated by subtracting these expected cases from the observed cases and dividing by the observed cases.

This procedure can be repeated for any dichotomous risk factor in the logistic regression model. It is also applicable when removing risk factors sequentially from the model and has been called 'sequential attributable fraction' [16]. However, when using the latter approach, the result is sensitive to the order of the risk factor removal from the model.

The dependence on the removal sequence can be simply addressed. If the risk factors are removed in every possible order and averaged over all obtained AFs, the average estimate does not depend on the order sequence anymore [16]. This approach has to be repeated *k*! times with *k *as the number of risk factors in the model. Eide calls this approach 'average attributable fraction' [16].

We provide codes for the software packages SAS, STATA and R to allow calculating average AFs from logistic regression [see Additional files 1, 2 and 3].

### Data

We used data of the National Health and Nutrition Examination Survey (NHANES) 2005–2006 to estimate AFs [7]. We focused on evidence based risk factors for cardiovascular disease. We restricted the study population to participants of at least 40 years of age who were not pregnant at the time of the investigation.

### Cardiovascular Disease (CVD)

Subjects were classified as having CVD according to their responses in the questionnaire on medical conditions. When subjects stated that a doctor or other health professional had told them having coronary heart disease, angina pectoris, or a heart attack, they were classified as having CVD.

We *a priori *considered smoking (more than 100 cigarettes ever), diabetes (physician told subject that he/she has diabetes), high total cholesterol level (physician told subject that he/she had high cholesterol level), low HDL cholesterol (< 45 mg/dl), and hypertension (systolic blood pressure > 140, diastolic blood pressure > 90, or a physician mentioned diagnosis of high blood pressure) as risk factors because of ample evidence from the literature and their previous inclusion in the Framingham risk score [17].

## Results

Data on 2,217 subjects aged 40 years and older with full information on CVD and respective risk factors were available. We restricted all analyses to this subset to ensure the same denominator in all analyses. There were 1,108 male subjects and 1,109 female subjects. A total of 1,179 (53%) subjects were 60 years and older.

Overall 279 (13%) subjects had evidence of CVD. The most frequent risk factor for CVD among the study population was smoking with 1,146 (52%) subjects who were classified as smokers. The least frequent risk factor was prevalent diabetes with 354 (16%) subjects affected. Frequencies of CVD and risk factors separated by age categories '40–59 years' and '60 and older' are shown in table 1.

**Table 1:** Description of dataset from the National Health and Nutrition Examination Survey 2005–2006 on 2,217 subjects of 40 years and older and full information on outcome CVD and displayed risk factors

	40 to 59 years (N = 1,038)		60 years and older (N = 1,179)	
Variable	n	%	n	%
CVD	52	5.0	227	19.3
Male	491	47.3	617	52.3
Hypertension	442	42.6	828	70.2
High total cholesterol	449	43.3	623	52.8
HDL cholesterol < 45 mg/dl	341	32.9	334	28.3
Smoking	495	47.7	651	55.2
Diabetes	113	10.9	241	20.4

The risk factor with the highest unadjusted individual risk for CVD was age of 60 years and older with an odds ratio of 4.5 (95% confidence interval: 3.3, 6.2) compared to subjects aged 40 to 59 years. This finding was also observed in multivariable logistic regression adjusting for other risk factors, yielding an odds ratio of 3.8 (95% confidence interval: 2.7, 5.1). Estimates for unadjusted and adjusted odds ratios for all risk factors are presented in table 2.

**Table 2:** Unadjusted and adjusted odds ratios (ORs) of risk factors for the outcome cardiovascular disease among 2,217 subjects of the NHANES dataset 2005–2006 aged 40 years and older.

Risk factor	Unadjusted OR	95% confidence interval	Adjusted^a ^OR	95% confidence interval
Age of 60 years and older	4.5	3.3, 6.2	3.7	2.7, 5.1
Male	2.0	1.6, 2.6	1.6	1.2, 2.1
Hypertension	2.8	2.1, 3.8	1.9	1.4, 2.6
High total cholesterol	2.1	1.6, 2.7	1.6	1.2, 2.1
HDL cholesterol < 45 mg/dl	1.9	1.5, 2.5	1.7	1.3, 2.2
Smoking	2.0	1.5, 2.6	1.7	1.3, 2.2
Diabetes	2.6	2.0, 3.5	1.9	1.4, 2.5

^a^adjusted for all risk factors displayed in the table

The AF for each risk factor considered was highly dependent on the method applied for its estimation. Hypertension, for example, appeared to account for 51% of all cases of CVD when applying the classical Levin's formula. When using adjusted odds ratios plugged into Levin's formula the AF was considerably reduced to 34%. However, the average AF directly derived from logistic regression after considering all permutations was only 16%. The variation between the different approaches was correspondingly high for other risk factors (table 3).

**Table 3:** Attributable fractions (AFs) of risk factors for the outcome cardiovascular disease among 2,217 subjects of the NHANES dataset 2005–2006 aged 40 years and older.

Risk factor	Levin	Adj OR Levin^a^	Bruzzi	Sequential AF^b^	Sequential AF^c^	Average AF	Average AF (not considering hypertension)
Age of 60 years and older	65.2	58.8	59.3	54.0	13.3	30.9	37.4
Male	33.6	22.8	24.1	10.5	6.6	9.8	10.2
Hypertension	50.9	33.6	36.2	11.9	15.3	15.7	-
High total cholesterol	33.8	23.0	24.4	5.5	12.7	10.0	12.5
HDL cholesterol < 45 mg/dl	21.8	17.3	17.7	3.2	10.6	7.1	8.0
Smoking	34.1	26.3	27.1	3.9	20.8	11.4	12.0
Diabetes	20.4	12.2	13.9	1.3	11.0	5.4	6.5
Sum	259.8	194.0	91.6^d^	90.3	90.3	90.3	86.6

^a^adjusted for all risk factors displayed in the table

^b^nota bene: These estimates are dependent on the order of the risk factors, in this case the order in which the variables are reported in the table.

^c^nota bene: These estimates are dependent on the order of the risk factors, in this case the opposite order in which the variables are reported in the table.

^d^Bruzzi's method allows calculating a summary AF that is not the sum of all individual AF.

The unadjusted AFs calculated using Levin's formula had a total sum of more than 200%. For estimates from the Levin formula using adjusted odds ratios from multivariable logistic regression the sum was 194% and also far above the possible maximum of 100%. The same applied for estimates according to the method suggested by Bruzzi, for which the estimates were comparable to estimates from Levin's formula considering adjusted odds ratios from logistic regression. However, this method also allows for calculating a summary AF that is not equivalent to the sum of all individual AFs and sums up to a number below 100% (table 3).

The sequential AFs were dependent on the order the risk factors were 'removed' from the study population. Results in the respective columns in table 3 were based on only two out of 7! = 5,040 possible permutations for k = 7 covariates. When firstly removing high age followed by gender, hypertension, high cholesterol, HDL-cholesterol, smoking and at last diabetes, the AF for age was the highest with 54% for age of at least 60 years and for diabetes was the lowest with 1% (table 3). In contrast, a model with inverse withdrawal of the risk factors yielded remarkably different estimates and e.g. the AF for age was only 13% for at least 60 years or older. However, the sum of AFs is always independent of the removal order and was 90% for the two different sequences.

Average AFs were considerably lower than unadjusted AFs from Levin's formula or estimates from Levin's formula with adjusted odds ratios from logistic regression (table 3).

The average AF for diabetes was 5.4% and was the lowest average AF observed for the risk factors considered. This contrasts with the individual risk of diabetes yielding an adjusted odds ratio of 1.9 (95% confidence interval: 1.4, 2.5), which was one of the highest among modifiable risk factors.

In an additional model not considering hypertension, the AF of smoking was similar to the model also considering smoking (table 3).

## Discussion

This study illustrates the use of AFs as an impact measurement of a risk factor on population level. Risk factors with similar odds ratios yielded quite different AFs indicating different impacts on population level by prevalence of risk factors. Unadjusted AFs tend to estimate higher AFs compared with adjusted estimates. Average AFs seem to provide the most plausible estimates of the approaches examined.

The results derived from the models are in accordance with the evidence for cardiovascular risk factors. Like others we observed cholesterol levels, hypertension, smoking and diabetes as important cardiovascular risk factors [2]. Our approach additionally allows assessing the impact of these risk factors on population level.

The approach with the most plausible results, the average AF has the advantage of not adding up to more than 100%. In contrast, the simple Levin approach often yields cumulative AFs of more than 100%. Some authors argue that this makes sense since an individual can have several risk factors and the disease can be therefore prevented in several ways [18]. However, if there are a considerable number of risk factors which are possibly correlated, it is obvious that unadjusted AFs from bivariate analyses may be biased providing an overestimation of the preventive potential and adjusted AFs should be rather considered.

Unfortunately there is no test statistic or other indicator of the appropriateness of a certain model including the covariates considered. The appropriateness of a model should be considered as regards content. The need to develop the 'most appropriate model' to investigate the research question, thus, remains the top priority since the overall AF and AFs of single risk factors possibly change after withdrawal of risk factors due to confounding or risk factors on the causal pathway. For example, hypertension as a risk factor for cardiovascular disease might be on the causal pathway of smoking related pathologies or confounded by smoking or an independent risk factor. Risk factors on the causal pathway of other considered risk factors should be omitted from respective models. To assess if hypertension is on the causal pathway of smoking similar decisions have to be made as in the estimation of individual risk factors. Since e.g. the AF for smoking status was similar in the model containing and not containing hypertension, hypertension does not seem to be exclusively on the causal pathway of the effect of smoking on cardiovascular disease.

Surprisingly, the approach of average AFs has only rarely been applied. The original article by Eide, published in 1995 [16], has been cited 46 times according to ISI web of knowledge (22^nd ^September 2008). Among these 44 citations there are 11 self-citations, 15 papers with methodological considerations, and only 20 articles applying the approach. The low number of applications might be due to the lack of inclusion of this method in statistical program packages. Therefore we provide functions/macros for commonly used statistical programs to encourage other researchers to calculate average AFs [see Additional files 1, 2 and 3].

### Methodological Considerations

The accuracy of AF estimates by the algorithms presented in this paper still depends on the completeness of the multivariable model. If important confounders are not considered in the model, an overestimation of AFs can occur similarly to an overestimation of individual risk factors in multivariable regression models. Other potential confounders might not be considered in our model leaving only 10% of cases for factors like heredity and all other environmental factors together. However, such a bias is only dependent on the number of covariates considered and not on the applied method.

The functions provided for calculating adjusted AFs in the appendix are based on logistic regression analysis. They do not allow for consideration of continuous explanatory variables within the model e.g. age in years. Consideration of continuous covariates is theoretically possible and is a matter of programming. However, an AF for a continuous variable might be difficult to communicate. Calculating an AF for the mean or an inter-quartile range of a continuous variable provides an estimate for a pre-defined but possibly arbitrary parameter change.

Although the approach of calculating AFs with the Levin formula and adjusted odds ratios from logistic regression has been shown to yield inconsistent estimates [5,19], we used this approach for illustration and comparison to other approaches and do not recommend it due to biased estimates.

The calculation of average AFs as discussed and favored in this paper requires access to original observational data. When using the method of average AFs in this paper, it is not possible to estimate adjusted average AFs by published aggregated data as for example in Levin's equation. Following this consideration, combined estimates from several studies (e.g. results from a meta-analysis) cannot be considered in average AFs as proposed in this paper. Although, such a combined estimate may be less subject to variation due to a higher sample size, such a combining of possibly biased estimates does not consider adjustment for confounding. Therefore, average AFs from original data remains important due to control for confounding even if only one data set is available.

The results generated from an adjusted AF model for a specific population may not be fitting to settings in other populations. This is likely to be due to varying prevalence of risk factors. Additionally, AFs in other populations may differ due to the impact of additional age or ethnic groups that were not included in the original sample. For inferences on population level analyses should be based on representative data from the population of interest.

## Conclusion

Preventive strategies in populations have to take into account the magnitude of targeted risk factors and their prevalence in the population for which the respective intervention is planned. The concept of average AFs provides a useful tool to address these issues. Application of simple formulas such as the Levin formula, however, may yield considerable overestimation of potential population impact of specific interventions. The estimation may be improved by the application of average AFs. Macros for the standard statistical software programmes are provided [see Additional files 1, 2 and 3]. Application of these formulae requires access to individual subject data.

## Competing interests

The authors declare that they have no competing interests.

## Authors' contributions

SR performed the statistical analyses, wrote the software code and wrote substantial parts of the manuscript. AMT wrote substantial parts of the manuscript and suggested the idea for the article. RvK was involved in writing the manuscript and revising it critically for important intellectual content. All authors read and approved the final manuscript.

## Pre-publication history

The pre-publication history for this paper can be accessed here:

http://www.biomedcentral.com/1471-2288/9/7/prepub

## Supplementary Material

Additional file 1 averageaf.sas. functions/macros for the SAS statistical software package. Click here for file

Additional file 2 averageaf.ado. functions/macros for the STATA statistical software package. Click here for file

Additional file 3 averageaf.r. functions/macros for the R statistical software package. Click here for file
